# Do driver’s characteristics, system performance, perceived safety, and trust influence how drivers use partial automation? A structural equation modelling analysis

**DOI:** 10.3389/fpsyg.2023.1125031

**Published:** 2023-04-17

**Authors:** Sina Nordhoff, Jork Stapel, Xiaolin He, Alexandre Gentner, Riender Happee

**Affiliations:** ^1^Department Transport and Planning, Delft University of Technology, Delft, Netherlands; ^2^Department Cognitive Robotics, Delft University of Technology, Delft, Netherlands; ^3^Toyota Motor Europe NV/SA, Brussels, Belgium

**Keywords:** partial automation, system performance, driver-initiated disengagements, perceived safety, trust, personality

## Abstract

The present study surveyed actual extensive users of SAE Level 2 partially automated cars to investigate how driver’s characteristics (i.e., socio-demographics, driving experience, personality), system performance, perceived safety, and trust in partial automation influence use of partial automation. 81% of respondents stated that they use their automated car with speed (ACC) and steering assist (LKA) at least 1–2 times a week, and 84 and 92% activate LKA and ACC at least occasionally. Respondents positively rated the performance of Adaptive Cruise Control (ACC) and Lane Keeping Assistance (LKA). ACC was rated higher than LKA and detection of lead vehicles and lane markings was rated higher than smooth control for ACC and LKA, respectively. Respondents reported to primarily disengage (i.e., turn off) partial automation due to a lack of trust in the system and when driving is fun. They rarely disengaged the system when they noticed they become bored or sleepy. Structural equation modelling revealed that trust had a positive effect on driver’s propensity for secondary task engagement during partially automated driving, while the effect of perceived safety was not significant. Regarding driver’s characteristics, we did not find a significant effect of age on perceived safety and trust in partial automation. Neuroticism negatively correlated with perceived safety and trust, while extraversion did not impact perceived safety and trust. The remaining three personality dimensions ‘openness’, ‘conscientiousness’, and ‘agreeableness’ did not form valid and reliable scales in the confirmatory factor analysis, and could thus not be subjected to the structural equation modelling analysis. Future research should re-assess the suitability of the short 10-item scale as measure of the Big-Five personality traits, and investigate the impact on perceived safety, trust, use and use of automation.

## 1. Introduction

SAE Level 2 partially automated driving has been implemented in passenger cars since 2015 combining Adaptive Cruise Control (ACC) and Lane Keeping Assist (LKA). Such systems automate braking, acceleration and lane keeping, while drivers are required to monitor the system whenever the automated driving features are engaged even if feet are off the pedals and the driver is not steering (SAE International, 2021).

Ample scientific studies provided evidence for trust predicting the behavioral intention to use automated cars (Kaur and Rampersad, 2018; Xu et al., 2018; Kettles and Van Belle, 2019; Zhang et al., 2020; Du et al., 2021; Benleulmi and Ramdani, 2022; Kenesei et al., 2022; Meyer-Waarden and Cloarec, 2022; Foroughi et al., 2023). Overtrust can lead to misuse, and undertrust can lead to disuse (Lee and Moray, 1994; Lee, 2008). Overtrust and misuse are key safety concerns for (current) lower automation levels. Undertrust and disuse can diminish the projected benefits of higher automation levels. In our recent interview study with 103 users of Tesla’s Autopilot and Full-Self-Driving (FSD) Beta system, overtrust in Level 2 capability was associated with eyes-off, mind-off and fatigued driving (e.g., drivers actively manipulating the steering wheel, and falling asleep behind the steering wheel with Autopilot engaged) (Nordhoff et al., 2023). Disuse refers to not using automation when it would, in fact, be beneficial (Lee, 2008). Drivers decided to disengage automation, and take back control in anticipation of system failure and lack of trust in the capability of the automation to safely execute a manoeuvre (Dixit et al., 2016; Gershon et al., 2021). Other reasons for disuse were driver’s general negative predispositions towards automation or annoyances caused by automation (‘bells and whistles’ principle), the need to disengage automation, false alarms, and low perceived reliability (De Winter et al., 2022).

It is commonly assumed that (perceived) safety of automated cars is a key requirement for acceptance (Osswald et al., 2012; Dixit et al., 2016; Pyrialakou et al., 2020; Cao et al., 2021; Manfreda et al., 2021). Scientific evidence supporting the role of (perceived) safety as direct predictor of acceptance and use of automated cars is inconclusive. In our previous study, perceived safety did not influence actual use of partial automation (Nordhoff et al., 2021), while in other studies it did influence the intention to use automated cars (Montoro et al., 2019; Detjen et al., 2020; Koul and Eydgahi, 2020).

Technology acceptance models, such as the Unified Theory of Acceptance and Use of Technology (UTAUT) (Venkatesh et al., 2012), assume that performance expectancy (or perceived usefulness) is a key factor impacting the intention to use and actual use of technology. This assumption is supported by scientific evidence showing that the (expected) benefits of automation related to safety, comfort, and efficiency are key drivers impacting the decision to use automated cars (Nordhoff et al., 2020).

Informed by the results of this literature review, the present study derives testable hypotheses as shown by Table 1.

**Table 1:** Hypothesis development – automation related.

Hypothetical path		Expected effect	Hypotheses
Independent variable	Dependent variable		
Trust	Use	+	Trust has a positive effect on use of partial automation.
	Perceived benefits	+	Trust has a positive effect on the perceived benefits of partial automation.
	Secondary task engagement	+	Trust has a positive effect on secondary task engagement.
Perceived safety	Use	+	Perceived safety has a positive effect on use of partial automation.
	Perceived benefits	+	Perceived safety has a positive effect on the perceived benefits of partial automation.
	Secondary task engagement	+	Perceived safety has a positive effect on secondary task engagement.
Perceived benefits	Use	+	Perceived benefits has a positive effect on use of partial automation.
Secondary task engagement		+	Secondary task engagement has a positive effect on use of partial automation.
Trust	Secondary task engagement	+	Trust has a positive effect on secondary task engagement.
Perceived safety		+	Perceived safety has a positive effect on secondary task engagement.

### 1.1. External variables impacting perceived safety and trust in partial automation

Technology acceptance models also assume that external variables (e.g., driver’s characteristics and system performance) influence the independent factors in the models (Nordhoff et al., 2016; Venkatesh et al., 2016). Driver’s characteristics including demographics and personality can influence trust in and perceived safety of partial automation, and thereby affect use of partial automation. Such relations are still poorly understood (see Modliński et al., 2022). In their theoretical model for trust in automated systems, Hoff and Bashir (2013) assumed that age, gender, and personality influence individual’s level of trust in automated systems. Experiments and surveys provided ambiguous empirical evidence on the relationship between age, gender, and trust in automation. It was found that trust in automated cars decreased (Dikmen and Burns, 2017), or increased with age (Zhang et al., 2020), or that the relationship between age and trust was not significant (Molnar et al., 2018). Yu et al. (2021) revealed that middle-aged drivers reported higher trust in Advanced Driver Assistance Systems (ADAS) than younger drivers. Moody et al. (2020) found that younger people were more likely to have favorable perceptions of safety of automated driving technology.

Males were more likely to report higher ratings of trust in and perceived safety of automated cars. Yu et al. (2021) observed that females had higher levels of trust in ADAS than males. Higher-educated people were more likely than lower-educated people to report more favorable perceptions of safety of automated driving technology (Moody et al., 2020).

Walker et al. (2018) showed substantial changes in driver’s trust ratings after an on-road experience of a partially automated car, with trust ratings decreasing after the experience. Metz et al. (2021) found that trust and perceived safety both increased after experiencing automated driving functions, with respondents spending more time on secondary tasks and less on monitoring the road ahead. In Montoro et al. (2019), driving experience (in years) and experience with driving crashes was positively associated with the perceived safety of automated vehicles. Cao et al. (2021) revealed that familiarity and driving experience correlated positively with the perceived safety of automated cars.

Previous research has shown that people’s attitudes and behavior are influenced by their personality (Devaraj et al., 2008). Personality is commonly captured by the ‘Big Five’ or OCEAN model, representing one of the most comprehensive and parsimonious personality measures (John and Srivastava, 1999; Lovik et al., 2017). The influence of personality on trust and perceived safety in partially automated cars is still poorly understood. Zhang et al. (2020) found a positive effect of the personality trait ‘openness’ on respondents’ trust in automated cars, while the personality trait ‘neuroticism’ had negative effects on trust in automated cars. Li et al. (2020) found a negative relationship between extraversion, openness, and trust in automated cars, respectively, while neuroticism, agreeableness, and conscientiousness did not have significant impacts on trust. Merritt and Ilgen (2008) found that extraverted people were more likely to have high trust in automated driving systems.

Studies have also shown that trust is a function of system performance. Choi and Ji (2015) found positive effects of system transparency, technical competence, and situation management on trust. Wilson et al. (2020) found that trust in partially automated cars was associated with the capability of automated cars to manage corners and regulate speed in response to other traffic. In the study of Dikmen and Burns (2017) drivers who experienced unexpected system performance were less likely to trust Autopilot.

Informed by the results of this literature review, the present study derives additional testable hypotheses as shown by Table 2.

**Table 2:** Hypothesis development – external variables.

Hypothetical path		Expected effect	Hypotheses
Independent variable	Dependent variable		
Age	Trust	-	Age has a negative effect on trust in partial automation.
	Perceived safety	-	Age has a negative effect on perceived safety of partial automation.
Gender (males)	Trust	+	Males are more likely to have report higher levels of trust in partial automation than females.
	Perceived safety	+	Males are more likely to report higher levels of perceived safety of partially automated cars than females.
Education	Trust	+	Education has a positive effect on trust in partial automation.
	Perceived safety	+	Education has a positive effect on perceived safety of partial automation.
Driving experience	Trust	+	Driving experience has a positive effect on trust in partial automation.
	Perceived safety	+	Driving experience has a positive effect on the perceived safety of partial automation.
Openness	Trust	+	Openness has a positive effect on trust in partial automation.
	Perceived safety	+	Openness has a positive effect on the perceived safety of partial automation.
Conscientious-ness	Trust	+	Conscientiousness has a positive effect on trust in partial automation.
	Perceived safety	+	Conscientiousness has a positive effect on the perceived safety of partial automation.
Agreeableness	Trust	+	Agreeableness has a positive effect on trust in partial automation.
	Perceived safety	+	Agreeableness has a positive effect on the perceived safety of partial automation.
Extraversion	Trust	+	Extraversion has a positive effect on trust in partial automation.
	Perceived safety	+	Extraversion has a positive effect on the perceived safety of partial automation.
Neuroticism	Trust	-	Neuroticism has a positive effect on trust in partial automation.
	Perceived safety	-	Neuroticism has a positive effect on the perceived safety of partial automation.
System performance	Trust	+	High system performance has a positive effect on trust in partial automation.
	Perceived safety	+	High system performance has a positive effect on the perceived safety of partial automation.

### 1.2. Research model and questions

**Figure 1:**
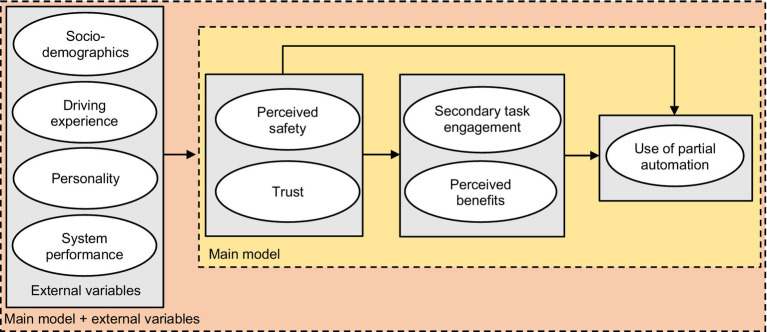
Research model shown for representational purposes.

The present study presents the research model in Figure 1.

Data on actual behavior and perception of drivers in partially automated cars is rare. Published studies are largely based on simulators rather than on-road pilots, collecting data from naïve and inexperienced respondents without sustained use of partial automation (De Winter et al., 2014; Montoro et al., 2019; Gershon et al., 2021). The present study probed the perception and behaviour of actual drivers of partially automated cars, through an online survey. We collected information of consumers with a substantial experience with partial automation, and asked them to reflect on their actual on-road behavior regarding automation use. In addition, we probed their perception to investigate trust and perceived safety, and other factors motivating automation use. The following three main research questions were addressed:

- To what extent do perceived safety and trust, perceived benefits, and secondary task engagement influence use of partial automation?
- To what extent do perceived safety and trust influence the perceived benefits of partial automation, and secondary task engagement?
- To what extent do driver’s characteristics (i.e., socio-demographics, driving experience, personality), and system performance influence perceived safety and trust in partial automation?

## 2. Method

### 2.1. Instrument and recruitment

To target current users of partially automated cars, we distributed the survey at Tesla’s supercharging stations near Utrecht, Dordrecht, and Amsterdam in the Netherlands in the form of a QR code. The link was further distributed in specialized communities and forums (e.g., Tesla Owners clubs and forums). Furthermore, the survey was shared in car-and mobility-related forums and groups of Reddit and Facebook, respectively. The authors of the present study further shared the link to the questionnaire on LinkedIn, and an anonymous link to access the questionnaire was sent to employees of Toyota Motor Europe using internal communication mailing.

The questionnaire was implemented using the questionnaire tool Qualtrics (www.qualtrics.com). The questionnaire instructions informed the respondents that it would take around 20 min to complete the questionnaire and that the work is organized by Delft University of Technology in the Netherlands. To improve data quality, Qualtrics applied several technologies preventing respondents from taking the survey more than once, detecting suspicious, non-human (i.e., bot) responses, and preventing search engines from indexing the survey.

### 2.2. Questionnaire content and measurement

#### 2.2.1. System functionality

Prior to participation in the questionnaire, respondents received a description about the functionality of partially automated cars to ensure that respondents had a sufficient understanding of partially automated cars. This description informed respondents that partially automated cars automate the acceleration, braking, and/or steering of the car. Furthermore, respondents were informed that partially automated cars have gas and brake pedals and a steering wheel and that they as human drivers have to supervise the performance of the car in order to resume manual driving. Their hands have to remain on or periodically touch the steering wheel, and their eyes remain on the road.

#### 2.2.2. Provision of written consent to participate in study

After respondents received the instructions, they were asked to provide their written consent to participate in the study. They were asked to declare that they have been informed in a clear manner about the nature and method of the research as described in the instructions at the beginning of the questionnaire. They were further asked to agree, fully and voluntarily, to participate in this research study. They were further informed that they retain the right to withdraw their consent and that they can stop participation in the study at any time. Finally, they were informed that their data will be treated anonymously in scientific publications, and will not be passed to third parties without their permission (Q1).

#### 2.2.3. Personal information and automation use

After respondents provided their written consent to participate in the study, they were asked to provide information about their age (Q2), gender (Q3), highest level of education completed (Q4), and personality (Q5–Q14). They were also asked to indicate access to a valid driver license (Q15), age of car (Q16), brand (Q17), car model (Q18), access to Lane Departure Warning (LDW), Lane Keeping Assist (LKA), and Adaptive Cruise Control (ACC) in their cars (Q19.1–Q19.3), and frequency of activating those systems (Q20.1–Q21.3). Those respondents who indicated that they had access to all three systems (i.e., LDW, LKA, and ACC) or a combination of two of the three systems were allowed to continue with the questionnaire. Respondents were asked to what extent the pandemic COVID–19 affected their mileage in the last 12 months as driver (Q21) and to select the number of kilometers/miles they drove in the last 12 months as driver (Q22). Respondents were also asked how often they used their partially automated car with speed and steering support (Q24). Respondents who did not indicate access to three of these systems or a combination of two of these were directed to the final questionnaire section on the evaluation of Human Machine Interfaces (HMIs) because it was expected that valid responses could be obtained for these questions without sustained use of automation. The questions measuring the evaluation of the HMIs will be subjected to further analysis in future studies. The personality questions were operationalized by the short 10-item measurement scale (Rammstedt and John, 2007), which was also applied in other studies (Kraus et al., 2020).

#### 2.2.4. Driving experience

With the next questions (Q23.1–Q23.7), respondents were asked to report the number of times they have been involved in risky driving-related situations in their lifetime as driver. These risky driving-related situations covered loss of concentration (Q23.1), minor loss of vehicular control while driving (Q23.2), driving fines (Q23.3), nearly having an accident (Q23.4), accident leading to material damage (Q23.5), accident in which the airbag was deployed (Q23.6), and accident leading to personal injury (Q23.7). The questions measuring the involvement in risky driving-related situations were taken from Deffenbacher et al. (2001); Delhomme et al. (2012); Disassa and Kebu (2019).

#### 2.2.5. System performance

Respondents were asked to indicate on a Likert scale from strongly disagree (1) to strongly agree (5) to what extent their partially automated car typically accelerates and decelerates smoothly (Q25), makes smooth, gentle steering corrections while they drive actively (Q26), detects lane markings on the roadway (Q27), detects the car ahead in their lane (Q28), starts to change lanes, and then returns to its lane halfway through the process (Q29), and asks them to take over control when they are not ready to take over control (Q30). The formulation of the questions was based on Reagan et al. (2020), while the questions Q29–Q30 were self-developed.

#### 2.2.6. Trust in partial automation

With the next section, respondents were asked to indicate on a scale from strongly disagree (1) to strongly agree (5) to what extent they trust their partially automated car to maintain speed and distance to the car ahead (Q31), and keeping the car centered in the lane (Q32) (Reagan et al., 2020). Next, respondents were asked to indicate to what extent they feel hesitant about activating the partially automated driving mode from time to time (Q33) (self-developed). Next, respondents were asked to rate to what extent they engage in other activities while driving their partially automated car (Q34) (Gold et al., 2015; Xu et al., 2018; Mason et al., 2020), and to what extent they always know when their car is in partially automated driving mode (Q35) (Körber, 2018). With the next self-developed questions, respondents were asked to rate to what extent the surrounding elements detected by their partially automated car are always clear to them (Q36), to what extent their partially automated car reminds them to take back full control (Q37), to keep their hands on the steering wheel (Q38), and to help them to keep using the partially automated car in the manner as advised by the manual (Q39). Next, respondents were asked to rate their trust in their partially automated car (Q40) (Choi and Ji, 2015). The next questions asked respondents to indicate to what extent they are unwilling to hand over control to their partially automated car from time to time (Q41), and to monitor the performance of their partially automated car most of the time (Q42).

#### 2.2.7. Secondary task engagement during manual and partially automated driving

With the next questions, respondents were asked to indicate how often they talk to fellow travelers (Q43.1 / Q44.1), watch videos or TV shows (Q43.2 / Q44.2), use the phone for calls (Q43.3 / Q44.3), texting (Q43.4/ Q44.4), music selection (Q43.5/ Q44.5), and setting/updating navigation (Q43.6/ Q44.6), reading text from book/phone (Q43.7/ Q44.7), eating and drinking (Q43.8/ Q44.8), monitoring the road ahead (Q43.9/ Q44.9), sleeping (Q43.10/ Q44.10), and engaging in other activities (Q43.11 / Q44.11) during both manual and partially automated driving. Questions Q43.11/Q44.11 were open-ended questions (“Other”), which were removed from the analysis of the present study.

#### 2.2.8. Perceived benefits

On a scale from strongly disagree (1) to strongly agree (5), respondents were asked to rate to what extent they use their partially automated car to reach their destination more safely (Q45), comfortably (Q46) (Nordhoff et al., 2020), pleasurable (Q47) (self-developed), and to use their time for other activities unrelated to driving (Q48) (Nordhoff et al., 2020).

#### 2.2.9. Driver-initiated disengagements

On a scale from strongly disagree (1) to strongly agree (5), respondents were further asked to indicate the reasons for disengaging partially automated driving when they notice they become sleepy (Q49), they do not trust it (Q50), they become bored (Q51), driving is fun (Q52), it is not necessary to use it (Q53), and when it is distracting or confusing (Q54). The questions Q49–Q52 were based on the studies of Lin et al. (2018) and Van Huysduynen et al. (2018) and the questions Q53–Q54 were self-developed.

#### 2.2.10. Perceived safety

Furthermore, respondents had to indicate to what extent they feel safe (Q55), relaxed (Q56), anxious (Q57) (Xu et al., 2018), and bored most of the time (Q58) when partially automated driving was engaged (Zoellick et al., 2019). With the next question, respondents were asked to indicate to what extent they are concerned about their general safety most of the time (Q59) (Xu et al., 2018). Next, respondents had to rate to what extent they entrust the safety of a close relative to their partially automated car (Q60) (Wien, 2019).

An ordinal Likert-scale was applied, and the order of the questions was randomized to prevent order effects.

### 2.3. Data analysis

The data was analyzed in three steps.

In the first step, descriptive statistics (i.e., means, standard deviations, and frequencies) of the questionnaire items that were subjected to the analysis of the present paper were computed.

In the second step, a confirmatory factor was conducted to estimate the measurement model, i.e., the measurement relations between the questionnaire items and their underlying latent constructs by assessing the internal consistency reliability, (i.e., Cronbach’s alpha), composite reliability, convergent validity, and discriminant validity. Convergent validity was assessed by the following four criteria: (1) Factor loadings should be significant, exceeding the recommended threshold of 0.60 on their scales; (2) Average variance extracted (AVE) should exceed the value of 0.50; (3) Construct reliability (CR) should exceed the threshold of 0.60; and (4) Cronbach’s alpha values should exceed the threshold of 0.60 (Hair, 2009).

Discriminant validity (i.e., uni-dimensionality of latent constructs) is established if the square root of the AVE of each latent construct exceeds the correlation coefficient between two latent constructs. The fit of the model was deemed acceptable if the Comparative Fit Index (CFI) and Tucker Lewis Index (TLI) ≥ 0.90, Root Mean Square Error of Approximation (RMSEA) ≤ 0.08, and the Standardized Root Mean Square Residual (SRMR) ≤ 0.06 (Hair, 2009).

In the third step, a structural equation modeling analysis was performed to assess the structural path relationships between the latent factors in the model. This step involves testing the structural path relationships between the latent factors in the model, reporting the standardized regression coefficients, standard error terms, significance levels, and variance accounted for in the latent constructs.

## 3. Results

### 3.1. Data filtering

Between November 24, 2020, and January 30, 2021, 1,557 questionnaires were completed. On average, respondents needed 86.12 min to complete the survey. Respondents who were identified as bots, who did not agree to participate in the study, did not have access to a valid driver license, reported to be younger than 18 years old, and who reported to never and less than monthly use their automated car with speed and steering support were removed from the analysis. In addition, only the responses from respondents reporting to have access to both ACC and LKA representing the functionality of partially automated cars, and who stated to own a car aged 4 years or less were subjected to the analysis given that partially automated driving was only introduced in 2015. *“I prefer not to respond”* and *“Not applicable to me”* responses were defined as missing values. 628 responses were maintained for the analysis.

### 3.2. Respondents

The profile of the respondents is presented in Table 3. 51% of respondents were between 36 and 55 years, and 81% of respondents were male. 77% of respondents reported to have a Bachelor or Master degree. 46% of respondents experienced loss of concentration, 23% nearly had an accident, and 19% received driving fines more than five times in their lifetime as driver. 93% of respondents never experienced accidents leading to personal injury, 90% accidents in which the airbag was deployed, and 36% accidents leading to material damage. 81% of respondents reported that they used their automated car with speed (ACC) and steering assist (LKA) at least 1–2 times a week, and 84 and 92% stated to activate LKA and ACC at least occasionally, respectively.

**Table 3:** Respondents’ profile (*M* = Means, SD = Standard deviation, *n* = number of respondents).

Questionnaire item	*M*	SD	Relative frequencies of response categories							
			0	1	2	3	4	5	6	n
Age (1 = 18–22, 2 = 23–35, 3 = 36–55, 4 = 56–69)	3.71	0.72	–	4%	33%	51%	12%	–	–	621
Gender (1 = Female, 2 = Male, 3 = Other)	–	–	–	81%	19%	–	–	–	–	628
Education (1 = High school diploma without apprenticeship / professional training, 2 = High school diploma with apprenticeship / professional training, 3 = Bachelor or Master degree, 4 = PhD or Dr. degree)	2.83	0.62	–	6%	11%	77%	6%	–	–	613
Age of car (in years) (1 = 0–1, 2 = 2–4)	1.22	0.41	–	78%	22%	–	–	–	–	592
Loss of concentration while driving (in lifetime as driver) (0 = 0, 1 = 1, 2 = 2, 3 = 3, 4 = 4, 5 = 5, 6 = 5+ events)	3.90	2.30	14%	7%	11%	11%	5%	6%	46%	587
Minor loss of vehicular control while driving (in lifetime as driver) (0 = 0, 1 = 1, 2 = 2, 3 = 3, 4 = 4, 5 = 5, 6 = 5+ events)	2.17	2.07	27%	20%	20%	11%	3%	4%	15%	602
Driving fines (in lifetime as driver) (0 = 0, 1 = 1, 2 = 2, 3 = 3, 4 = 4, 5 = 5, 6 = 5+ events)	2.44	2.18	25%	16%	19%	11%	6%	4%	19%	603
Nearly having an accident (in lifetime as driver) (0 = 0, 1 = 1, 2 = 2, 3 = 3, 4 = 4, 5 = 5, to 6 = 5+ events)	2.73	2.18	17%	19%	19%	12%	5%	4%	23%	601
Accident that led to material damage (in lifetime as driver) (0 = 0, 1 = 1, 2 = 2, 3 = 3, 4 = 4, 5 = 5, 6 = 5+ events)	1.35	1.51	36%	28%	19%	9%	4%	2%	4%	609
Accident in which the airbag was deployed (in lifetime as driver) (0 = 0, 1 = 1, 2 = 2, 3 = 3, 4 = 4, 5 = 5, 6 = 5+ events)	0.15	0.54	90%	8%	1%	1%	0%	0%	0%	610
Accident that led to personal injury (in lifetime as driver) (0 = 0, 1 = 1, 2 = 2, 3 = 3, 4 = 4, 5 = 5, 6 = 5+ events)	0.11	0.51	93%	5%	1%	1%	0%	0%	0%	610
Actual use of partially automated cars										
Frequency of using automated car with speed and steering support (1 = Never, 2 = Less than monthly, 3 = Less than weekly but more than once a month, 4 = 1–2 times a week, 5 = 3–4 times a week, 6 = At least five times a week)	4.82	1.36	–	2%	7%	11%	15%	21%	45%	606
Frequency of activation of LKA (Lane Keeping Assist) (1 = Never, 2 = Rarely, 3 = Occasionally, 4 = Frequently, to 5 = Always)	3.83	1.22	–	6%	10%	17%	29%	38%	–	623
Frequency of activation of ACC (Adaptive Cruise Control) (1 = Never, 2 = Rarely, 3 = Occasionally, 4 = Frequently, to 5 = Always)	3.98	0.93	–	3%	4%	14%	49%	29%	–	623

A Bachelor or Master degree was treated equivalent to having a college degree.

#### 3.2.1. Driver-initiated disengagements

**Figure 2:**
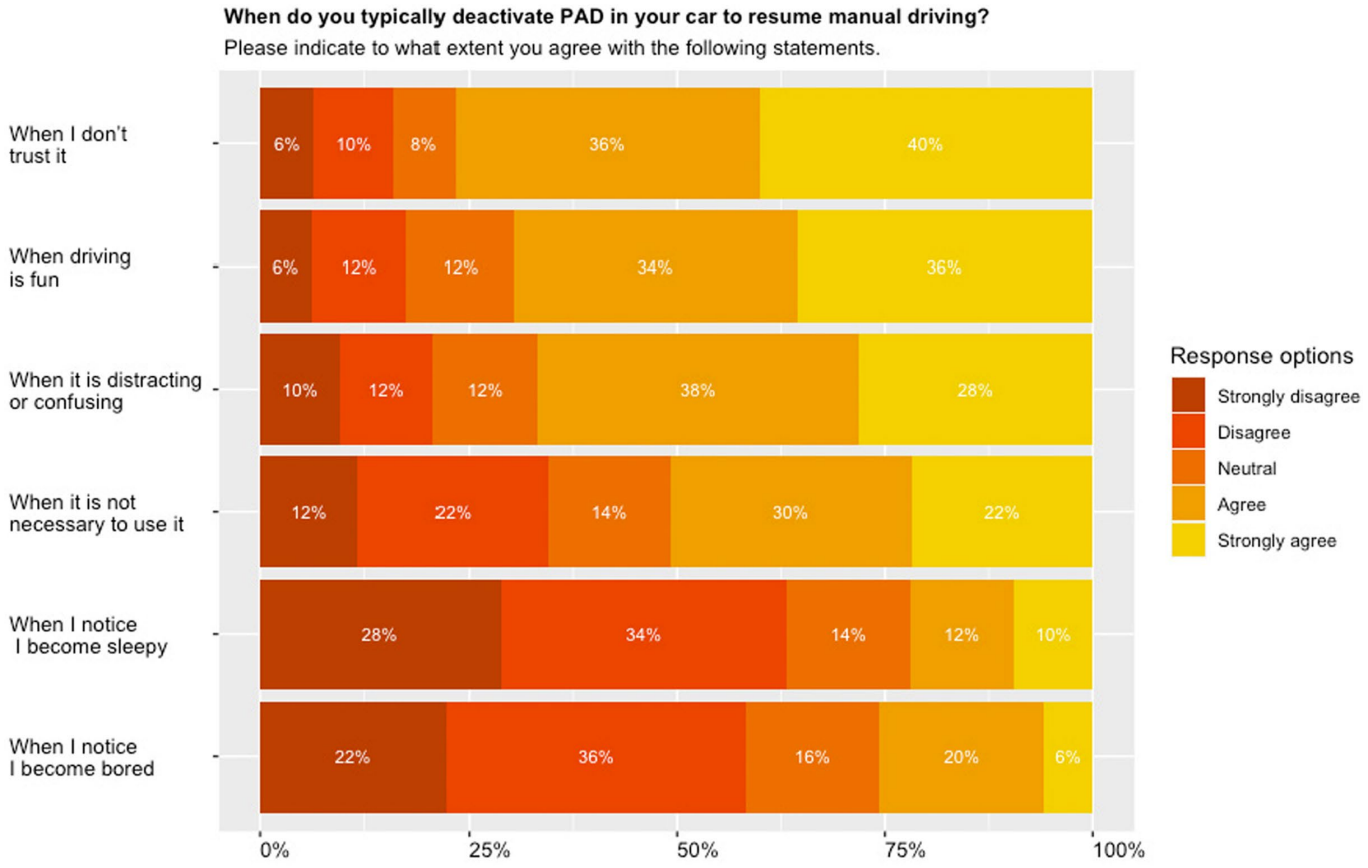
Driver-initiated disengagement of partial automation; stacked bar plots presenting relative proportions of questions pertaining to disengaging partial automation sorted from highest (top) to lowest (bottom).

Figure 2 shows that 76% of respondents reported to disengage partial automation when they do not trust it (***M*** = 3.94, SD = 1.20), and 68% when driving is fun. Only 22% of respondents disengaged the system when respondents noticed they become sleepy (***M*** = 2.40, SD = 1.28), and 26% when they noticed they become bored (***M*** = 2.51, SD = 1.20).

#### 3.2.2. System performance

**Figure 3:**
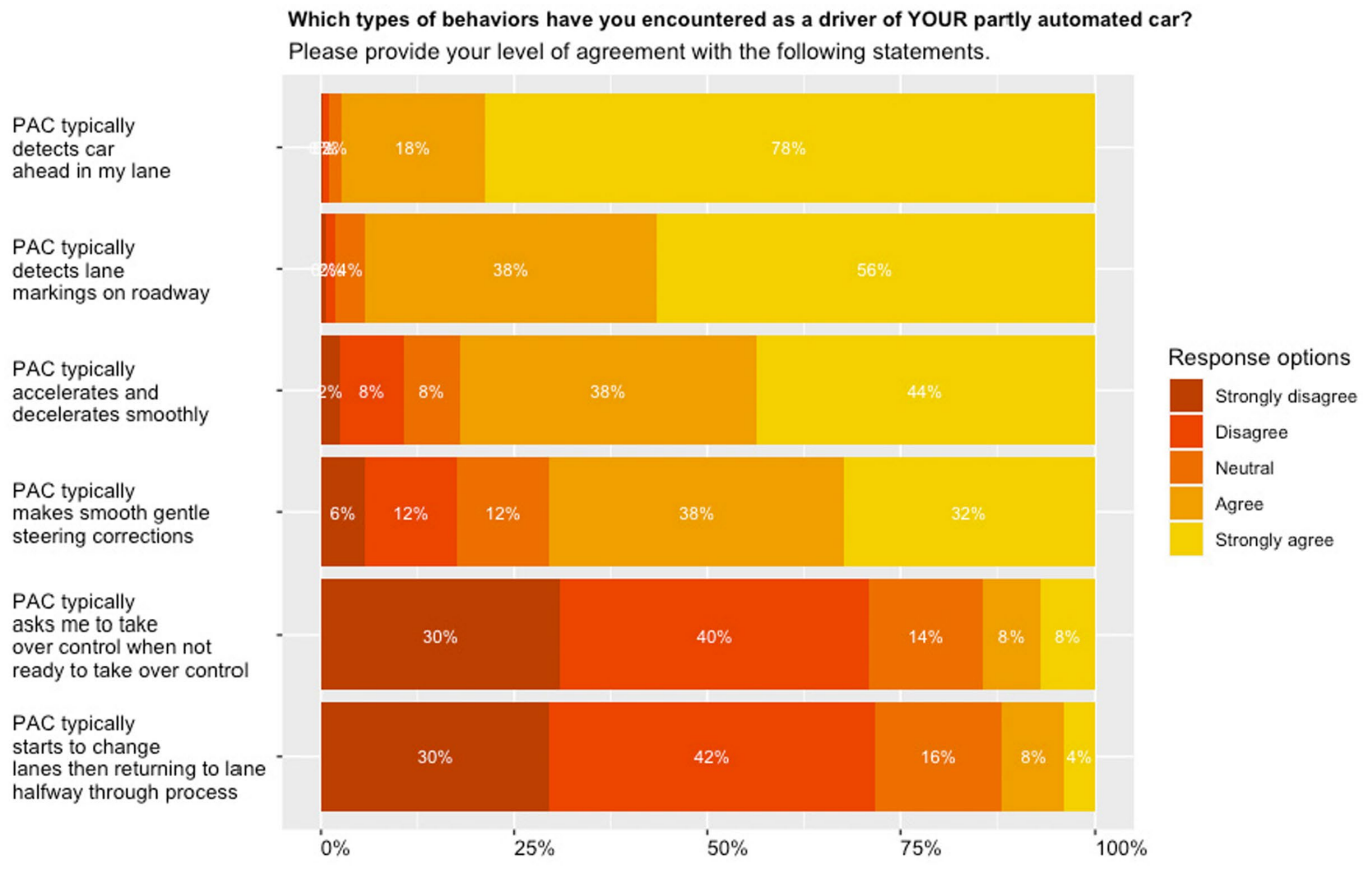
System performance; stacked bar plots presenting relative proportions of questions pertaining to system performance sorted from highest (top) to lowest (bottom).

As shown by Figure 3, respondents provided the strongest agreement with the partially automated car detecting the car ahead in their lane (***M*** = 4.75, SD = 0.54), the lane markings on the roadway (***M*** = 4.48, SD = 0.69), with 96 and 94% indicating their agreement with these questions. The weakest agreement was obtained for the partially automated car typically starting to change lanes and then returning to its lane halfway throughout the process (***M*** = 2.15, SD = 1.06), with 16 and 12% of respondents agreeing with these questions.

### 3.3. Confirmatory factor analysis

A confirmatory factor analysis was conducted to estimate the relationships between the latent constructs and their observed variables (i.e., questionnaire items). Table 4 shows the standardized factor loadings for all constructs after omitting questionnaire items from the analysis whose loadings did not meet the recommended threshold of 0.60. From the five personality dimensions only neuroticism and extraversion proved to be valid and reliable. The loadings of the indicator variables of the four remaining personality constructs were lower than the recommended threshold of 0.60, and thus dropped from the analysis. As two of the three items loading on driver engagement were below 0.60, the latent construct driver engagement was dropped from the analysis. Cronbach’s alpha and composite reliability was larger than 0.60 for all constructs, which demonstrates sufficient internal consistency reliability of the constructs. The average variance extracted (AVE) exceeded the recommended threshold of 0.50 for all constructs except for extraversion (0.493), and trust (0.459). As shown by Table 5, the square root of the AVE for all constructs was larger than the Spearman correlation coefficients between the constructs, which demonstrates that the latent constructs are sufficiently distinct (discriminant validity). The fit of the measurement model was acceptable, with the indexes exceeding the recommended thresholds [Comparative Fit Index (CFI) = 0.954, Tucker Lewis Index (TLI) = 0.934, Root Mean Square Error of Approximation (RMSEA) = 0.050]. The *χ*^2^ test statistic [*χ*^2^/df (degrees of freedom)] was 2.286, falling below the recommended threshold of <2.5.

**Table 4:** Confirmatory factor analysis (*M*, means, SD, standard deviation, *ƛ*, lambda, *⍺*, Cronbach’s alpha, CR, Composite reliability, AVE, Average variance extracted).

Latent variable	Observed variable	*M*	SD	*ƛ*	*⍺*	CR	AVE	Source
Openness (O)	O1: Someone who has an active imagination	4.13	0.86	Omitted from analysis due to factor loading <0.60				Rammstedt and John (2007)
	O2: Someone who has few artistic interests (reverse-scaled)	3.04	1.17					
Conscientiousness (C)	C1: Someone who tends to be lazy (reverse-scaled)	3.32	1.16	Omitted from analysis due to factor loading <0.60				
	C2: Someone who does a thorough job	4.34	0.73					
Extraversion (E)	E1: Someone who is reserved (reverse-scaled)	2.77	1.07	0.61	0.65	0.65	0.49	
	E2: Someone who is outgoing and sociable	3.52	1.09	0.78				
Agreeableness (A)	A1: Someone who is generally trusting	3.87	0.98	Omitted from analysis due to factor loading <0.60				
	A2: Someone who tends to find fault with others (reverse-scaled)	3.07	1.06					
Neuroticism (N)	N1: Someone who gets nervous easily	2.64	1.11	0.78	0.67	0.66	0.51	
	N2: Someone who is relaxed handles stress well	2.24	0.98	0.62				
Trust (TRU)	TRU1: I trust my PAD to maintain speed and distance to the car ahead	4.49	0.77	0.65	0.72	0.72	0.46	Based on Reagan et al. (2020)
	TRU2: I trust my PAD to keep the car centered in the lane	3.96	1.08	0.64				
	TRU3: I can trust my PAC	4.01	0.95	0.74				Based on Choi and Ji (2015)
Perceived safety (PS)	PS1: I feel safe most of the time	4.26	0.73	0.78	0.82	0.82	0.56	Xu et al. (2018)
	PS2: I feel relaxed most of the time	4.09	0.85	0.88				
	PS3: I feel anxious most of the time (reverse-scaled)	4.08	0.93	0.67				
Driver engagement (DE)	DE1: The surrounding elements detected by my PAC are always clear to me	3.85	1.03	Self-created	Omitted from analysis due to factor loadings <0.60			Self-created
	DE2: My PAC always reminds me to keep my hands on the steering wheel	4.28	1.08					
	DE3: My PAC helps me to keep using it in the manner as advised by the manual	4.08	0.91					
Secondary task engagement (STE)	STE1: I use my PAC because it helps me to use my time for other activities unrelated to driving	2.19	1.16	0.73	0.70	0.70	0.54	Based on Nordhoff et al. (2020)
	STE2: I engage in other activities while driving my PAC	2.29	1.20	0.74				Based on Mason et al. (2020)
Perceived benefits (PB)	PB1: I use my PAC because it helps me to reach my destination more comfortably	4.42	0.86	0.75	0.71	0.70	0.54	Based on Nordhoff et al. (2020)
	PB2: I use my PAC because it makes driving more pleasurable	4.15	1.02	0.73				Self-created
Actual use (AU)	AU1: Frequency of using partially automated driving with speed and steering control	4.82	1.35	0.68	0.71	0.73	0.53	Based on Nordhoff et al. (2020)
	AU2: Frequency of activation of ACC	3.98	0.93	0.83	0.83			

**Table 5:** Inter-construct correlation matrix.

	*N*	*E*	TRU	STE	PB	PS	AU
*N*	0.714						
*E*	-0.228***	0.702					
TRU	-0.118**	0.021	0.677				
STE	0.030	0.047	0.182***	0.735			
PB	-0.082*	0.006	0.522***	0.158***	0.736		
PS	-0.347***	0.043	0.480***	0.115**	0.377***	0.772	
AU	-0.127***	-0.026	0.361***	0.235***	0.386***	0.340***	0.725

*N*, Neuroticism; E, Extraversion; TRU, Trust; STE, Secondary task engagement; PB, Perceived benefits; PS, Perceived safety; AU, Actual use; ***p < 0.001, **p < 0.01, *p < 0.05, all other correlations are not significant.

### 3.4. Structural equation modelling analysis

A structural equation modelling analysis was conducted to assess how:

- Perceived safety, trust, perceived benefits, and secondary task engagement influence use of partial automation;
- Perceived safety and trust influence secondary task engagement, and the perceived benefits of partial automation;
- Driver’s characteristics (i.e., socio-demographics, driving experience, personality), and system performance influence perceived safety and trust in partial automation.

The structural equation model consisted of valid and reliable latent constructs identified in the confirmatory factor analysis, and single-item indicators measuring driving experience, system performance, and disengaging partial automation. These items did not form valid and reliable composite scales in the confirmatory factor analysis. The use of single item measures in structural equation models is acceptable if single item variables measure specific aspects or behaviors (Hair, 2009)—a condition that is met in the present study. The results of the structural equation modelling analyses are presented in Tables 6, 7, and will be discussed in the subsequent section.

**Table 6:** Structural equation modelling results (i.e., *β* = standardized beta coefficients, significance level = *** *p* < 0.001, **p < 0.01, *p < 0.05, all other correlations are not significant, *R*^2^ = variance explained), main model.

Hypothetical path		*β* and significance level
Independent variable	Dependent variable	
Trust	Actual use	0.435*
	Disengaging when driver becomes sleepy	0.007
	Disengaging when driver becomes bored	-0.098
	Disengaging when driving is fun	-0.071
	Disengaging when not necessary to use	-0.253**
	Disengaging when distracting to use	-0.174*
	Perceived benefits	0.729***
	Secondary task engagement	0.309**
Perceived safety	Actual use	0.020
	Disengaging when driver becomes sleepy	-0.094
	Disengaging when not trusting it	0.056
	Disengaging when driver becomes bored	-0.069
	Disengaging when driving is fun	0.104
	Disengaging when not necessary to use	0.002
	Disengaging when distracting to use	-0.019
	Perceived benefits	0.118
	Secondary task engagement	-0.015
Perceived benefits	Actual use	0.065
Secondary task engagement		0.130
Trust	Secondary task engagement	0.309**
Perceived safety		-0.015
*R* ^2^		
Secondary task engagement	0.090	
Perceived benefits	0.649	
Actual use	0.306	
Model fit parameters		
CFI	0.942	
TLI	0.912	
RMSEA	0.053	
SRMR	0.044	
*χ*^2^/df	1.955	

**Table 7:** Structural equation modelling results (i.e., *β* = standardized beta coefficients, significance level = ***p < 0.001, **p < 0.01, *p < 0.05, all other relations are not significant, *R*^2^ = variance explained), main model + external variables.

Hypothetical path		*β* and significance level
Independent variable	Dependent variable	
Age	Trust	-0.041
	Perceived safety	0.012
Gender (males)	Trust	0.072
	Perceived safety	0.235***
Education	Trust	-0.006
	Perceived safety	0.030
Loss of concentration while driving	Perceived safety	-0.014
Minor loss of vehicular control while driving		0.067
Driving fines		0.062
Nearly having an accident		0.039
Accident leading to material damage		-0.080
Accident with deployment of airbag		0.001
Accident leading to personal injury		0.060
Loss of concentration while driving	Trust	0.012
Minor loss of vehicular control while driving		-0.092
Driving fines		-0.109
Nearly having an accident		0.027
Accident leading to material damage		0.066
Accident with deployment of airbag		-0.148
Accident leading to personal injury		0.257***
Driving mileage	Trust	0.131*
	Perceived safety	-0.004
Extraversion	Trust	-0.079
	Perceived safety	-0.036
Neuroticism	Trust	-0.181*
	Perceived safety	-0.412***
PAD typically accelerating and decelerating smoothly	Trust	0.325***
PAD typically making smooth and gentle steering corrections		0.090
PAD typically detecting lane markings on roadway		0.383***
PAD typically detecting car ahead in lane		0.124*
PAD typically starting to change lanes, and then returning to lane halfway throughout its process		-0.073
PAD typically asking me to take over control when not ready to take over control		-0.041
PAD typically accelerating and decelerating smoothly	Perceived safety	0.268***
PAD typically making smooth and gentle steering corrections		0.013
PAD typically detecting lane markings on roadway		0.220***
PAD typically detecting car ahead in lane		−0.006
PAD typically starting to change lanes, and then returning to lane halfway throughout its process		−0.090
PAD typically asking me to take over control when not ready to take over control		−0.022
Trust	Actual use	0.466**
	Disengaging when driver becomes sleepy	0.013
	Disengaging when driver becomes bored	−0.066
	Disengaging when driving is fun	0.012
	Disengaging when not necessary to use	−0.213**
	Disengaging when distracting or confusing to use	−0.139*
	Perceived benefits	0.564***
	Secondary task engagement	0.309**
Perceived safety	Actual use	0.237
	Disengaging when driver becomes sleepy	−0.103
	Disengaging when not trusting it	0.101
	Disengaging when driver becomes bored	−0.153*
	Disengaging when driving is fun	0.042
	Disengaging when not necessary to use	−0.142
	Disengaging when distracting or confusing to use	−0.196**
	Perceived benefits	0.364***
	Secondary task engagement	0.120
Perceived benefits	Actual use	−0.082
Secondary task engagement		0.071
Trust	Secondary task engagement	−0.044
Perceived safety		0.120
*R* ^2^		
Trust	0.545	
Perceived safety	0.401	
Secondary task engagement	0.013	
Perceived benefits	0.591	
Actual use	0.289	
Model fit parameters		
CFI	0.828	
TLI	0.796	
RMSEA	0.050	
SRMR	0.062	
*χ*^2^/df	1.602	

The hypotheses specifying the relationships between the personality dimensions ‘openness’, ‘conscientiousness’, and ‘agreeableness’ were not tested given that they did not form valid and reliable scales in the confirmatory factor analysis.

## 4. Discussion

This study presents results of an online questionnaire with respondents with actual extensive experience with SAE Level 2 partially automated cars. The main objectives were to investigate how driver’s individual characteristics (i.e., socio-demographics, driving experience, personality), system performance, perceived safety and trust in partial automation influence use of partial automation.

### 4.1. Descriptive statistics

The analysis of descriptive statistics revealed that regarding system performance, the strongest agreement was obtained for the partially automated car detecting the car ahead in their lane. A weaker agreement was obtained for the partially automated car accelerating and decelerating smoothly. Both responses relate to ACC where apparently detection is rated higher than smooth control. The second-highest mean rating was obtained for detecting the lane markings on the roadway, while a weaker agreement was found for the partially automated car making smooth and gentle steering corrections. These responses relate to LKA where again, detection is rated higher than smooth control. The weakest agreement was obtained for the partially automated car typically starting to change lanes and then returning to its lane halfway throughout the process, and for asking respondents to take over control when they are not ready to take over control, with 12% and 16% of respondents (strongly) agreeing with the corresponding questions.

Our study revealed that the number one reason for driver-initiated disengagements was a lack of trust in the system, while sleepiness was a minor reason for driver-initiated disengagements: 76% of respondents agreed (strongly) with disengaging due to a lack of trust, while only 22% agreed (strongly) to disengage when sleepy and 62% even disagreed (strongly) to disengage when sleepy. Van Huysduynen et al. (2018) and Wilson et al. (2020) revealed that respondents most frequently disengaged automation due to a lack of trust in the partially automated car. Lack of trust pertained to the ability of the automation to handle merging manoeuvres, to recognize an obstacle, to handle a situation in poor weather conditions, or because respondents felt uncomfortable travelling too close to another vehicle (Wilson et al., 2020). Other studies associated a lack of trust with technology disuse (Lewandowsky et al., 2000; Choi and Ji, 2015; Lee et al., 2021). Disengaging Tesla’s Autopilot system in situations when drivers became sleepy has been reported by Van Huysduynen et al. (2018). However, in our study only 22% report to disengage partially automated driving when sleepy. This represents a serious safety concern and future studies should investigate which ‘sleepiness level’ (e.g., mind-wandering, nodding off, sleeping) represents a safety concern that diminishes driver performance in critical take-over situations.

### 4.2. Structural equation modelling analysis

The structural equation modelling analysis provided evidence to support several study hypotheses in Tables 1, 2. The analysis revealed that trust had a positive impact on driver’s propensity for secondary task engagement. This corresponds with previous research, which has shown that overtrust in automation was associated with unsafe secondary task engagement during partially automated driving (Casner et al., 2016; Endsley, 2017; Banks et al., 2018; Lin et al., 2018; Casner and Hutchins, 2019; Wilson et al., 2020; Kim et al., 2021). We also found a strong impact of trust on the factor ‘perceived benefits’, showing that an increase in trust in partial automation increases the likelihood to appreciate the perceived benefits of partial automation. It is interesting to note that perceived safety did not influence secondary task engagement. Among the three factors – perceived benefits, secondary task engagement, and trust – only trust did influence actual use of partial automation. Previous research has shown that the perceived benefits of automation strongly influenced the intention to use automated cars (which is regarded as one of the most important predictors for actual use) (Nordhoff et al., 2020).

The structural equation modelling analysis demonstrated a small impact of gender on perceived safety, with males reporting higher levels of perceived safety than females. This aligns with Moody et al. (2020) where males had more favorable perceptions of safety than females. The impact of age on perceived safety and trust was not significant. Other studies found mixed results, where Zhang et al. (2020) found a positive relation between age and trust, whereas in (Moody et al., 2020; Best et al., 2021), younger people had more optimistic safety perceptions than elderly people. Our study findings about age and gender are in line with research investigating attitudes towards automation, which has provided ambiguous evidence about the role of gender and age on key beliefs (e.g., perceived usefulness, ease of use, intention to use) towards automated cars. A common conception held in industry is that users of (automated) cars should be targeted based on key socio-demographic attributes (Christensen et al., 2022). Future research should critically re-assess the role of age and gender, using a representative and balanced sample of the general population of car drivers, considering other personality traits being pivotal for the human-machine collaboration and acceptance of partial automation (e.g., locus of control) (e.g., see Syahrivar et al., 2021).

Our study found a small positive effect of the frequency of experiencing accidents leading to personal injury on trust. This finding is plausible as respondents with severe experiences with accidents are more likely to appreciate the safety benefits of automated cars, and/or may even use these cars to be safer, both objectively and subjectively. Studies have shown more positive (safety) perceptions among individuals being involved in risky driving behavior and accidents (Turner and McClure, 2003; Holland and Hill, 2007; Montoro et al., 2019). It is plausible that accidents with partially automated cars contribute to lower trust in partially automated cars. Lee et al. (2021) revealed that failure of automation contributed to a decrease in trust, which, however, was quickly rebuilt. We also found a positive effect of driving mileage on trust, which shows that experience has a positive effect on trust in automation.

We found a small negative effect of neuroticism on perceived safety, and a moderate effect on trust in partial automation. Likewise, Zhang et al. (2020) revealed that neuroticism had a negative effect on trust in automated cars. The results of the confirmatory factor analysis supported the validity and reliability of two of five personality dimensions: extraversion and neuroticism. This corresponds with the study of Kraus et al. (2020), which reported low Cronbach’s alpha levels for openness, conscientiousness, and agreeableness. Studies applying a longer-item scale to measure the Big-Five personality dimensions produced internally consistent and valid personality dimensions (Braitman and Braitman, 2017; Niranjan et al., 2022).

The study revealed that the ACC, and LKA performance of partial automation did impact respondent’s perceived safety and trust in partially automated cars. This implies that enhancing the reliability and possibly the intuitiveness of the automation controlling the longitudinal and lateral part of the driving task may be an effective means to influence perceived safety and trust in automation. Research has shown that respondents were more comfortable when the automated car had a more defensive driving style, which was characterized by lower speed, and smoother accelerations and decelerations (Beggiato et al., 2020). It should be studied which driving style of the automated vehicle can produce calibrated levels of perceived safety and trust, matching the actual capabilities and limitations of the system.

We also studied the specific conditions for disengaging the system. When trust was high, the likelihood to disengage the system when it was not necessary to use, and when it was distracting or confusing to use decreased. This may imply that respondents with high levels of trust may have a higher level of technological understanding and knowledge of where and when to use automation. We also found that when perceived safety was high, the propensity to disengage the system when it was distracting or confusing, and when drivers noticed they become bored was lower. It should be assessed to what extent boredom during partially automated driving represents a safety-critical physiological state that may compromise driver’s safety, e.g., take-over performance.

### 4.3. Limitations and future research

First, researchers and respondents may not be able to clearly discriminate between perceived safety and trust. This is illustrated by the labelling of the construct ‘trust in safety’ (Kettles and Van Belle, 2019; Morrison and Belle, 2020), and the measurement of perceived safety by items pertaining to trust in automation (Koul and Eydgahi, 2020). Perceived safety is closely related to perceived risk, and can even be used interchangeably. Perceived risk is generally defined as the level of risk experienced by users of driving automation (He et al., 2022). Finally, risk is also closely related to trust (Hoff and Bashir, 2015; Lazanyi and Maraczi, 2017; Zhang et al., 2019; Tenhundfeld et al., 2020). We recommend future research to re-assess the operationalization of perceived safety and trust in automation.

Second, studies should consider the development of personality scales tailored to the context of road vehicle automation, and investigate the impact on perceived safety, trust, and automation use. The items of existing personality scales such as the Big5 are stated in very generic terms, making the direct transfer to the context of automation challenging.

Third, we also recommend future research to integrate self-efficacy (i.e., individual’s beliefs in own capabilities to produce certain effects) (Bandura and Wessels, 1994), and privacy concerns in models with personality, perceived safety, trust, and automation use. These factors may gain in importance with greater connectivity in automated cars (Duboz et al., 2022). In the study of Zhang et al. (2019) privacy concerns were not associated with trust in automated cars. In another study, privacy concerns had negative effects on trust in automated cars (Meyer-Waarden and Cloarec, 2022), and neurotic individuals were less comfortable with data transmitting (Kyriakidis et al., 2015). Du et al. (2021) found that respondents with higher perceived capabilities to use automated cars had higher trust in automated cars.

Fourth, through on-road observation studies, future research should also relate subjective feelings of safety to more objective indicators measuring perceived safety or risks, e.g., unexpected system disengagements, false alarms (Bauchwitz and Cummings, 2020), and frequency of driver-initiated disengagements.

Fifth, future research should perform validation checks, e.g., by assessing drivers’ knowledge about these systems, to verify whether the system drivers said that they had were available in their cars. It cannot be ruled out that respondents misunderstood our descriptions of the system functionality of partially automated driving.

Sixth, our study is one of the few studies surveying actual users of SAE Level 2 partially automated cars. As the present study mainly used U.S. American communities and forums, the dataset is not fully representative of the European population of drivers of partially automated cars. Respondents may well have an above average interest in and enthusiasm for automated driving (Nordhoff et al., 2021). It is also possible that respondents engaged in socially desirable response behavior, being overly positive to address the challenges associated with partially automated driving, especially Tesla Autopilot (Banks et al., 2018; Lin et al., 2018; Casner and Hutchins, 2019; Wilson et al., 2020). Future research should investigate perceived safety, trust and automation use among a more balanced and representative sample of European car drivers.

### 4.4. Conclusion

The present study surveyed actual extensive users of SAE Level 2 partially automated cars to investigate how driver’s characteristics (i.e., socio-demographics, driving experience, personality), system performance, perceived safety and trust in partial automation influence use of partial automation. People reporting a higher driving mileage had a higher level of trust in partially automated cars, while the effect on perceived safety was not significant. Neuroticism was negatively related to perceived safety, but not to trust in partial automation. System performance (ACC and LKA functionality) influenced perceptions of safety and trust in partial automation. Trust also influenced disuse of partial automation in situations when the system was not necessary, distracting or confusing to use. Respondents positively rated the performance of Adaptive Cruise Control (ACC) and Lane Keeping Assistance (LKA). ACC was rated higher than LKA and detection of lead vehicles and lane markings was rated higher than smooth control for ACC and LKA, respectively. Respondents reported to primarily disengage (i.e., turn off) partial automation due to a lack of trust in the system and when driving is fun. They rarely disengaged the system when they noticed they become bored or sleepy. Structural equation modelling revealed that trust had a positive effect on secondary task engagement during partially automated driving, while the effect of perceived safety on secondary task engagement was not significant. Regarding driver’s characteristics, we did not find a significant effect of age on perceived safety and trust in partial automation. Neuroticism had a negative effect on perceived safety and trust, while extraversion did not impact perceived safety and trust.

## Data availability statement

The raw data supporting the conclusions of this article will be made available by the authors, without undue reservation.

## Ethics statement

Ethical review and approval was not required for the study on human participants in accordance with the local legislation and institutional requirements. The patients/participants provided their written informed consent to participate in this study.

## Author contributions

SN: conceptualization, methodology, software, validation, formal analysis, investigation, resources, data curation, writing—original draft, writing—review and editing and visualization. JS: writing—review and editing. XH: writing—review and editing. AG: writing review and editing. RH: writing—review and editing, supervision, project administration, funding acquisition. All authors contributed to the article and approved the submitted version.

## Funding

This research was conducted as part of the project “Investigating Trust in Automation,” co-funded by Toyota Motor Europe NV/SA. The funders had no role in data analysis, decision to publish, or preparation of the manuscript.

## Conflict of interest

AG was employed by Toyota Motor Europe NV/SA.

The remaining authors declare that the research was conducted in the absence of any commercial or financial relationships that could be construed as a potential conflict of interest.

## Publisher’s note

All claims expressed in this article are solely those of the authors and do not necessarily represent those of their affiliated organizations, or those of the publisher, the editors and the reviewers. Any product that may be evaluated in this article, or claim that may be made by its manufacturer, is not guaranteed or endorsed by the publisher.
